# The Age of the Soft-Girl Era: How Public Health Scholars May Seize Opportunity of Innovatively Promoting Reproductive Health and Nutritional Health Among Black Women of Color

**DOI:** 10.1089/heq.2024.0063

**Published:** 2025-01-08

**Authors:** Vanessa Nicholson-Robinson

**Affiliations:** Public Health and Community Medicine, Boston, Massachusetts, USA.

**Keywords:** African American, health equity, methods, nutrition, reproductive health, FIM (food is medicine)

## Abstract

In the current soft-girl era, a soft lifestyle promotes living with ease, comfort, healing, and joy. As health equity programs evolve, they should provide safe spaces for participants’ experiences, desires, and motivations for wellness living. Contributions of the soft-girl era movement challenge the notions for historically marginalized women to thrive in their health rather than merely surviving through it. If public health fields are to expand, including the fields of Black maternal health and Black food justice, Black participation is critical. The movement offers researchers to acknowledge participant voice, thereby gaining their trust, interest, and on-going participation in health programs.

## Introduction to the Soft-Life

Living a soft life is a practice within the soft girl era movement that has become a cultural trend within female audiences over the past decade. The soft-girl era lifestyle may be integrated in public health programs when researchers establish a more standardized definition of the “soft-life.” There are, however, a few challenges. The first challenge is that research on soft-lifestyle living is limited in regard to its impact on public health programs. This article is among the first, if not the first, to promote the study of how the applicability of soft living may potentially aid researchers in recruiting, retaining, and refining data from Black female participants involved in their health interventions. The second challenge is that the definition of soft-lifestyle living is ever-evolving and heavily dependent upon a community’s experiences, up-bringing, and culture. For example, it cannot be assumed that all communities involving women of color think the same; therefore, their wants and needs will vary across populations. The soft-girl era presents an opportunity to investigate not just what desires Black women have, but how they would want their desires to be met. Soft life promotes the conscious action of individuals and communities investing more into themselves compared with what they had invested before the adoption of the lifestyle. It is a matter of choosing the path of least resistance along with the absence of constant anxiety, stress, and hustle culture.^1–3^

## Sojourner Syndrome

Black women are historically familiar with being forced to live life through a survival lens. Despite improvements in addressing health disparities, they are still left at the bottom of the social ladder in American society. The Sojourner Syndrome describes social identities of African American women based on historical referents and adaptive behaviors that fostered survival and resilience under oppressive circumstances.^4^ Within the Sojourner framework, weathering is a specific component forcibly practiced by Black women. Weathering posits that Blacks experience early health deterioration because of the cumulative impact of repeated experience with social or economic adversity and political marginalization.^5^ Practical applications of public health focus on disease prevention, disease reduction, and overall optimal health. The social movement of the soft-girl era aligns with these same practical applications of public health via the promotion of physical stress reduction and mental health support for women. Simultaneously, the soft-girl era opposes the Sojourner Syndrome regarding practical applications of public health. The foundational component of the Sojourner Syndrome is restlessness. Such an activity overtime may lead to preventable stress-related chronic illnesses and an overall reduced quality of life. Contrastingly, the soft-girl era shifts to a place of ease and comfort where women have the autonomy to be respected, seen, heard, and ultimately thrive to achieve optimal health.

## African American Perspectives of Public Health Programs

Black female participation is critical to inform on best research practices and improve women’s health for this population. African Americans, however, are the least likely to begin participation and/or complete participation in public health programs—many of which address chronic health and maternal health.^6–7^ Mistrust is a primary factor that helps to explain barriers. One study specifically mentions that mistrust among African Americans was particularly associated with their perception that research will benefit Whites or the research institution and not people of color. Furthermore, mistrust related to the fear of purposeful mistreatment and experimentation was often characterized as being treated like a lab rat or guinea pig. Lastly, mistrust with signing the informed consent was related to the perception that individuals are relinquishing their rights and instead providing the researcher with legal protection against any harm that may be inflicted on participants.^8^ Community voice helps to inform on misalignment and gaps in regard to recruitment and retention methods for this population. Some practical ways proven to address mistrust involve (1) training local residents to serve as health ambassadors, (2) building bridges across sectors, and (3) establishing transparency in partnerships.^9^ Based on the foundational concepts of living the soft life, researchers may seize the current moment of the soft-girl era by translating methods that align with practices of safety, honesty, and respect to participant voice, where it may have been previously overlooked in the past programs regarding Black women audiences.

## Potential to Address Gaps Within the Black Maternal Health Space via the Soft-Girl Era

Centers for Disease Control and Prevention data confirmed that 85% of maternal deaths are preventable.^10^ The same information also provides hope in that we have the power to change this narrative for Black birthing moms and young Black girls who may become future mothers. Researchers should partner with their population of interest as opposed to disregarding their experiences. This action aligns with the soft-girl era’s emphasis on rest, trust, and healing. They must also include those on the team that culturally represent their population of interest and place them in positions of leadership. The Florida longitudinal infant mortality study (1992–2015) demonstrates how culturally representative team members positively impacted the health of Black birthing moms. For instance, when newborns were born to Black mothers who were cared for by Black providers, the mortality they suffered, as compared to Whites, was halved.^11^ Study findings suggest further research is necessary to explore how perceived trust and peace of mind from cultural concordance may help to address maternal health disparities.

Another suggested way to align the soft-girl era lifestyle with optimal birth outcomes for Black women is by prioritizing their access to doulas. Doula care may not be offered or accessible to parents in need, causing stress and potential harm.^12^ Doula care, however, empowers patients by allowing them to express their values and preferences, and advocate for their needs with comfort and ease. These characteristics are similar to the goals of what has been affectionately known among Black women as practicing living the “soft-life” lifestyle. Community-based doulas, for example, can provide culturally and linguistically congruent care that supports communication between mothers and their health care team. Doula care has also proven to lower rates for preterm birth and decrease the risk of cesarean birth as it provides continual emotional and relational support through birth.^13–14^ This is a factor identified by Black women’s health organizations as an essential aspect of the birthing experience.^15^

## Potential Applicability of Soft Girl Life within Nutritional Health

Addressing the nutritional health needs of U.S.-born Black women is essential to their chronic health, as four out of five African American women are classified as being overweight or obese.^16^ People who are overweight are more likely to suffer from a host of chronic health challenges, including high blood pressure, high levels of blood fats, diabetes, and low-density lipoprotein (LDL) cholesterol. All of these health outcomes further place Black women at higher risk for heart disease and stroke.^17^ Black birthing mothers may be particularly vulnerable, as they are more prone to outcomes of developing type 2 diabetes after being diagnosed with gestational diabetes and preeclampsia in comparison to their non-Black racial counterparts.^17,18^

Within the era of soft lifestyle living, motivational interviewing may be used as a complimentary method to encourage program participants to practice mindful eating. Motivational interviewing has been found to be useful for strengthening the motivation for behavioral change in patients with various behaviorally influenced health problems and for promoting treatment adherence.^19^ The common factor shared between soft life living and the motivational interviewing method is that both meet at the intersection of practicing and considering healthy self-prioritization and overall well-being. Another method that could be potentially cohesive with soft-lifestyle living is through the implementation of what is known as “Food is Medicine” (FIM) programs. FIM programs, for example, recognize that access to high-quality nourishment is essential for well-being.^20^ By addressing nutritional needs within the context of providing access to high-quality fresh produce, FIM interventions play an important role in preventing chronic illnesses faced by Black women of color. There are, however, challenges for researchers to consider. FIM interventions emphasize nutritious food being made available for everyone. Black women are part of a marginalized community of people who commonly face issues relating to food injustice in forms of food inaccessibility and food inequity. It is not enough to merely understand the desires of what Black women want to eat, but just as importantly, their desires of how nutritious food is available to them in a way that supports their comfort, ease, healing, and joy. As researchers implement FIM interventions in marginalized communities, a plan for how desires are addressed should be identified before beginning their investigations. The suggested approach may be implemented as a pilot study to identify best practice methodologies prior to serving a larger audience. There is currently no data that investigates the relationship between FIM Interventions and concepts derived from soft lifestyle living. It is, therefore, worthy to explore the relationship to examine a new method of assessing food interests and challenges to food access. Soft lifestyle living allows for the understanding of how to center communities of Black women by holding space for them to express and partner with program interventionists to ensure a healthy, balanced eating lifestyle.

## Abbreviations Used

CDC: Centers for Disease Control and Prevention
FIM: Food is Medicine
LDL: Low-Density Lipoprotein
